# Preliminary Clinical Observations Following Multiwavelength Diode Laser Treatment for Facial Rejuvenation: A Three‐Patient Case Series

**DOI:** 10.1111/jocd.71259

**Published:** 2026-10-27

**Authors:** Jesse Chong Jia Xi, Kyu‐Ho Yi

**Affiliations:** ^1^ Dream Clinic Kuala Lumpur Malaysia; ^2^ You and I Clinic Seoul Republic of Korea

**Keywords:** case series, erythema, facial rejuvenation, multiwavelength diode laser, pigmentation, skin tightening

## 1 Introduction

Nonablative facial rejuvenation aims to improve visible skin characteristics while minimizing epidermal disruption and recovery time. Clinical concerns may include skin laxity, facial contour, pigmentation, erythema, and textural irregularities. Previous studies have investigated diode and Nd:YAG laser systems for facial skin tightening and remodeling, providing a rationale for exploring nonablative laser‐based rejuvenation approaches [1, 2, 3, 4, 5].

A combined 755‐, 810‐, and 1064‐nm laser system has previously been investigated for nonablative skin rejuvenation. Jo et al. reported clinical improvement and histological increases in collagen and elastic bundles following serial treatments [1]. Earlier research using an 810‐ and 940‐nm diode laser also reported improvements in facial skin tightening [2]. Because optical absorption and scattering vary with wavelength and tissue properties, combining wavelengths may influence the distribution of deposited energy [6, 7]. However, the actual penetration depth and thermal distribution depend on device configuration and treatment parameters. Depth‐specific heating cannot be assumed from wavelength selection alone and was not directly investigated in the present case series.

Nonablative laser treatments have been investigated for several aesthetic outcomes, including erythema reduction and dermal remodeling. Histological evidence of new collagen formation has been reported following nonablative 1064‐nm Nd:YAG laser treatment [3]. Experimental research in a mouse model of rosacea‐like skin lesions has also demonstrated changes in erythema and collagen‐related markers following 1064‐nm laser exposure [8]. Nevertheless, these findings were obtained using different laser systems or experimental conditions and cannot establish equivalent biological effects for the multiwavelength diode protocol used in the present study.

This retrospective case series describes clinical observations in three patients who underwent multiwavelength diode laser treatment for facial aesthetic concerns. The objective was to describe the treatment protocols and available photographic and skin‐analyzer findings, identify relevant confounding factors, and discuss the limitations of the available evidence. The study was descriptive and was not designed to establish treatment efficacy or demonstrate a depth‐specific thermal mechanism.

## 2 Materials and Methods

This retrospective descriptive case series was prepared from an existing clinical case presentation containing clinical photographs, treatment protocols, and three‐dimensional (3D) skin‐analyzer outputs. Three adult patients were included, representing different aesthetic indications: facial rejuvenation with erythema and pigmentation, jawline contouring, and nasolabial fold and jawline conditioning. Patient characteristics, treatment courses, and relevant confounding factors are summarized in Table 1.

**TABLE 1:** Patient characteristics, treatment courses, and potential confounding factors.

Case	Patient	Clinical indication	Treatment course	Potential confounder
1	48‐year‐old woman; Fitzpatrick IV	Facial rejuvenation; erythema; pigmentation; nasolabial folds	3 sessions at 4‐week intervals	Polynucleotide injections after each session
2	28‐year‐old woman; Fitzpatrick IV	Jawline contouring; facial slimming; nasolabial folds	1 session	None reported in supplied material
3	38‐year‐old man; Fitzpatrick II	Facial rejuvenation; nasolabial folds; jawline	3 sessions at 4‐week intervals	Calcium hydroxylapatite filler approximately 5 months earlier

The available clinical material was reviewed descriptively. No control group was included, and the treatment effects were not evaluated using a comparative study design.

The treatments were described in the source material as being performed with a multiwavelength diode laser system incorporating 755‐, 810‐, and 1064‐nm wavelengths. The treatment protocol consisted of two steps: stacked irradiation followed by dynamic in‐motion irradiation.

During the first step, the reported fluence was 7 J/cm^2^ per stack, with five stacks and a nominal cumulative fluence of 35 J/cm^2^. This cumulative value represents the arithmetic sum of five 7‐J/cm^2^ exposures and assumes that all five exposures were delivered to the same treatment area. During the second step, dynamic in‐motion irradiation was performed at a reported fluence of 5–7 J/cm^2^.

The reported energy distribution differed between the treatment protocols. For Cases 1 and 3, the stacked step delivered a recorded 10 kJ per facial side, and the dynamic step delivered a recorded 20 kJ per facial side. The reported total energy per visit was therefore 60 kJ, calculated as (10 + 20) × 2 kJ. Each patient underwent three sessions at 4‐week intervals.

For Case 2, the stacked step delivered a recorded 8 kJ per facial side, followed by 12 kJ per facial side during the dynamic step. The reported total energy was therefore 40 kJ, calculated as (8 + 12) × 2 kJ. This patient underwent one treatment session.

These calculations explain the arithmetic relationship between the recorded energy values for the two treatment steps and the reported total energy per visit. However, the available documentation did not establish whether the step‐specific energy values were obtained from device‐displayed measurements or another recording method. The relationship between cumulative energy and the reported fluence could not be independently verified because the treatment area, output power, and exposure duration were not documented.

The recorded treatment parameters are summarized in Table 2. Manufacturer and model information, wavelength‐specific output power, emission mode, simultaneous or sequential wavelength delivery, beam dimensions, handpiece configuration, scanning speed, pass overlap, and cooling specifications were not available in the supplied clinical material. These omissions limit the technical reproducibility of the treatment protocol.

**TABLE 2:** Recorded multiwavelength diode laser treatment parameters.

Parameter	Cases 1 and 3 (three‐session protocol)	Case 2 (single‐session protocol)
Wavelengths	755, 810, and 1064 nm	755, 810, and 1064 nm
Treatment sessions	3	1
Treatment interval	4 weeks	Not applicable
Step 1 technique	Stacked irradiation	Stacked irradiation
Fluence per stack	7 J/cm^2^	7 J/cm^2^
Number of stacks	5	5
Nominal cumulative fluence	35 J/cm^2^ ^a^	35 J/cm^2^ ^a^
Step 1 reported energy per facial side	10 kJ	8 kJ
Step 2 technique	Dynamic in‐motion irradiation	Dynamic in‐motion irradiation
Step 2 reported fluence	5–7 J/cm^2^	5–7 J/cm^2^
Step 2 reported energy per facial side	20 kJ	12 kJ
Reported total energy per visit	60 kJ	40 kJ

*Note:* The reported energy per visit is the sum of the recorded step‐specific energies for two facial sides: (10 + 20) × 2 = 60 kJ for Cases 1 and 3; (8 + 12) × 2 = 40 kJ for Case 2. These arithmetic totals do not independently verify the optical energy delivered by the device.

^a^ The nominal cumulative fluence of 35 J/cm^2^ equals five exposures of 7 J/cm^2^ only if they were delivered to the same area. Actual spatial dose distribution and the relationship of fluence to total energy could not be verified. “Not documented” denotes information absent from the supplied material.

Clinical outcomes were assessed descriptively using available before‐and‐after clinical photographs. Three‐dimensional skin‐analyzer outputs were available for Case 1 and included redness and pigmentation scores, quantities, areas, and percentages. These device‐specific measurements were used to describe observed changes rather than to establish clinical efficacy.

The recorded skin‐analyzer measurements are summarized in Table 3. The available clinical material did not establish exact posttreatment assessment intervals or a uniform follow‐up schedule across all three patients. No comparative statistical analysis was performed.

**TABLE 3:** Three‐dimensional skin‐analyzer measurements in Case 1 before and after three sessions.

Parameter	Before treatment	After three sessions
Redness score	42.73	54.50
Mild redness quantity	522	380
Moderate redness quantity	682	444
Severe redness quantity	253	185
Mild redness area	20.89	18.60
Moderate redness area	18.95	13.66
Severe redness area	4.44	2.43
Mild redness percentage	17.33%	16.77%
Moderate redness percentage	15.34%	12.02%
Severe redness percentage	3.50%	2.13%
Pigmentation score	22.97	35.29
Mild pigmentation quantity	1420	990
Moderate pigmentation quantity	1125	770
Severe pigmentation quantity	393	241
Mild pigmentation area	23.32	22.90
Moderate pigmentation area	12.80	8.50
Severe pigmentation area	4.65	1.59
Mild pigmentation percentage	18.75%	20.14%
Moderate pigmentation percentage	10.18%	7.38%
Severe pigmentation percentage	3.64%	1.39%

*Note:* Values were transcribed from the supplied 3D skin‐analysis table. The scoring system is device‐specific; the clinical meaning of higher overall scores was not established in the source. Units for area measurements were not specified. Mild pigmentation percentage increased, whereas moderate and severe pigmentation percentages decreased. Case 1 also received polynucleotide injections after each laser session.

## 3 Case Presentations

### 3.1 Case 1: Anti‐Aging, Redness, Pigmentation, and Nasolabial Folds

A 48‐year‐old woman with Fitzpatrick skin Type IV underwent treatment for facial rejuvenation, erythema, pigmentation, and nasolabial fold concerns. The treatment consisted of three multiwavelength diode laser sessions performed at 4‐week intervals, with a reported total energy of 60 kJ per visit.

Polynucleotide injections were administered after each laser session. Polynucleotides have previously been investigated for aesthetic applications, including improvements in skin quality [9, 10]. Because laser treatment and polynucleotide injections were combined, their individual contributions to the observed outcomes could not be distinguished.

Following three treatment sessions, the available clinical photographs suggested improvements in facial tonal evenness, visible erythema, pigmentation irregularity, and nasolabial fold appearance (Figure 1).

**FIGURE 1:**
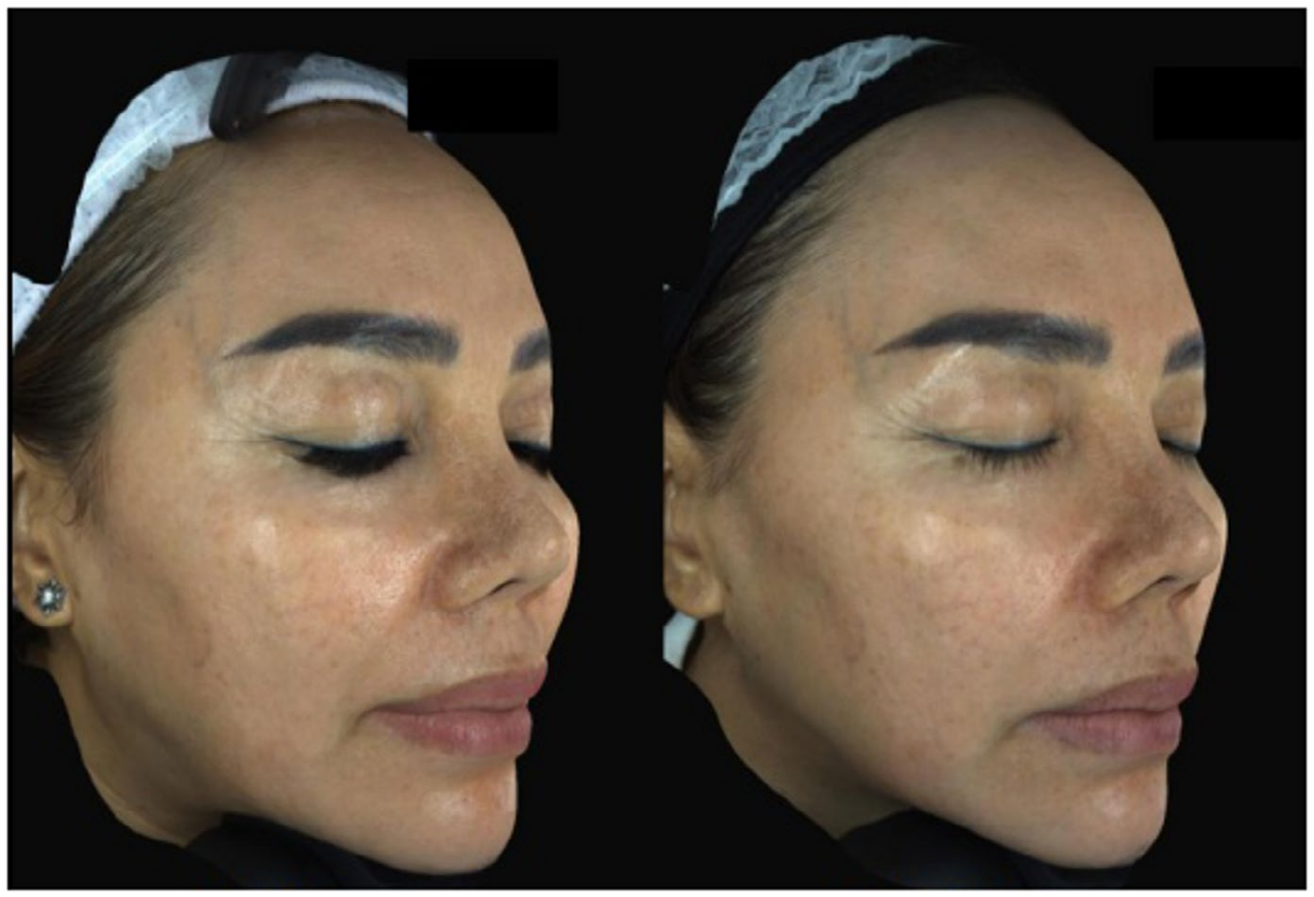
Case 1: Clinical photographs obtained before treatment and after three sessions of multiwavelength diode laser treatment combined with polynucleotide injections. The images provide a qualitative comparison of facial erythema, tonal evenness, and nasolabial fold appearance.

The three‐dimensional skin‐analyzer outputs demonstrated changes in several recorded redness and pigmentation parameters (Figure 2; Table 3).

**FIGURE 2:**
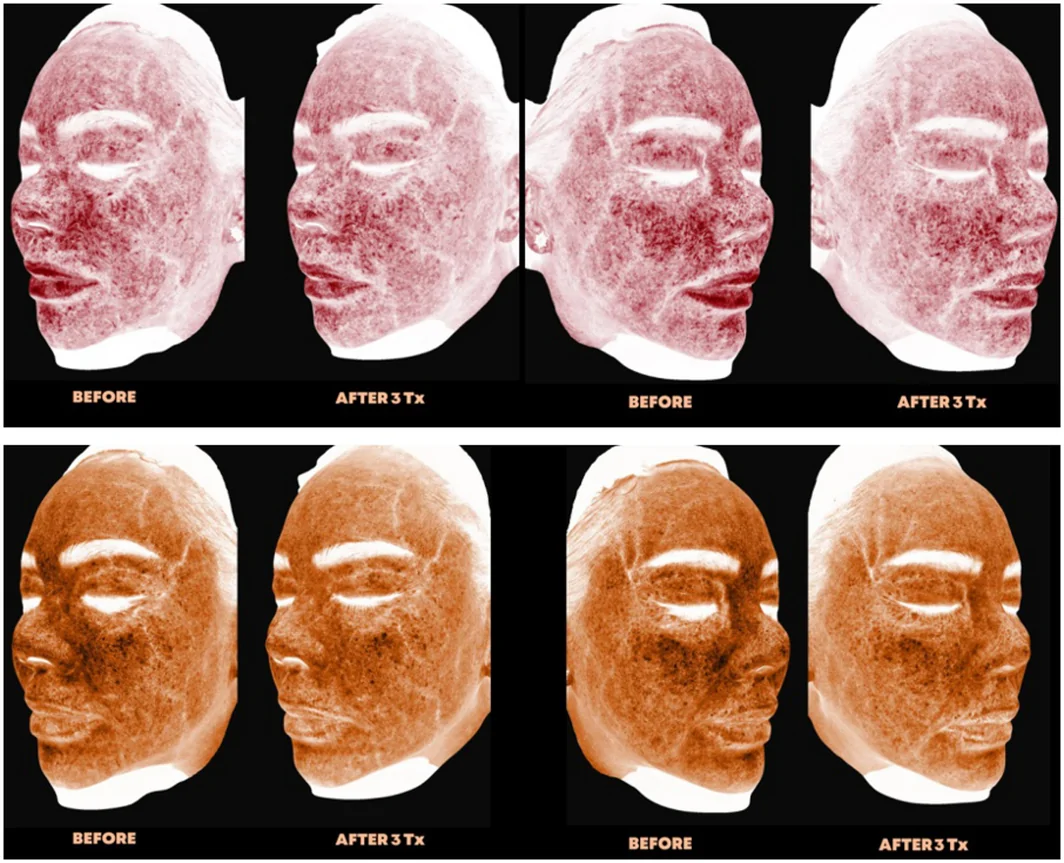
Case 1: Three‐dimensional skin‐analyzer maps illustrating the available redness and pigmentation measurements before treatment and after three treatment sessions. Changes in the recorded parameters are described in the text and summarized in Table 3.

The redness score increased from 42.73 to 54.50. Redness quantities for mild, moderate, and severe categories decreased from 522, 682, and 253 to 380, 444, and 185, respectively. The corresponding redness areas decreased from 20.89, 18.95, and 4.44 to 18.60, 13.66, and 2.43. Mild, moderate, and severe redness percentages changed from 17.33%, 15.34%, and 3.50% to 16.77%, 12.02%, and 2.13%, respectively.

The pigmentation score increased from 22.97 to 35.29. Pigmentation quantities for mild, moderate, and severe categories decreased from 1420, 1125, and 393 to 990, 770, and 241, respectively. The corresponding pigmentation areas changed from 23.32, 12.80, and 4.65 to 22.90, 8.50, and 1.59. Mild pigmentation percentage increased from 18.75% to 20.14%, whereas moderate and severe pigmentation percentages decreased from 10.18% and 3.64% to 7.38% and 1.39%, respectively.

These findings represent descriptive changes in device‐specific measurements. The clinical meaning and direction of the overall analyzer scores were not established in the supplied material, and the measurement units for the reported area parameters were not specified. The observed changes cannot be attributed exclusively to the laser because polynucleotide injections were administered after each session.

### 3.2 Case 2: Jawline Contouring, Facial Slimming, and Nasolabial Fold Reduction

A 28‐year‐old woman with Fitzpatrick skin Type IV underwent one multiwavelength diode laser session for jawline contouring, facial slimming, and nasolabial fold concerns.

The reported total energy was 40 kJ, comprising 8 kJ per facial side during stacked irradiation and 12 kJ per facial side during dynamic irradiation.

Comparison of the available clinical photographs suggested a more defined jawline, reduced apparent cheek fullness, and a softer nasolabial fold transition following treatment (Figure 3). These observations were qualitative, and no objective measurements of facial volume or contour were available.

**FIGURE 3:**
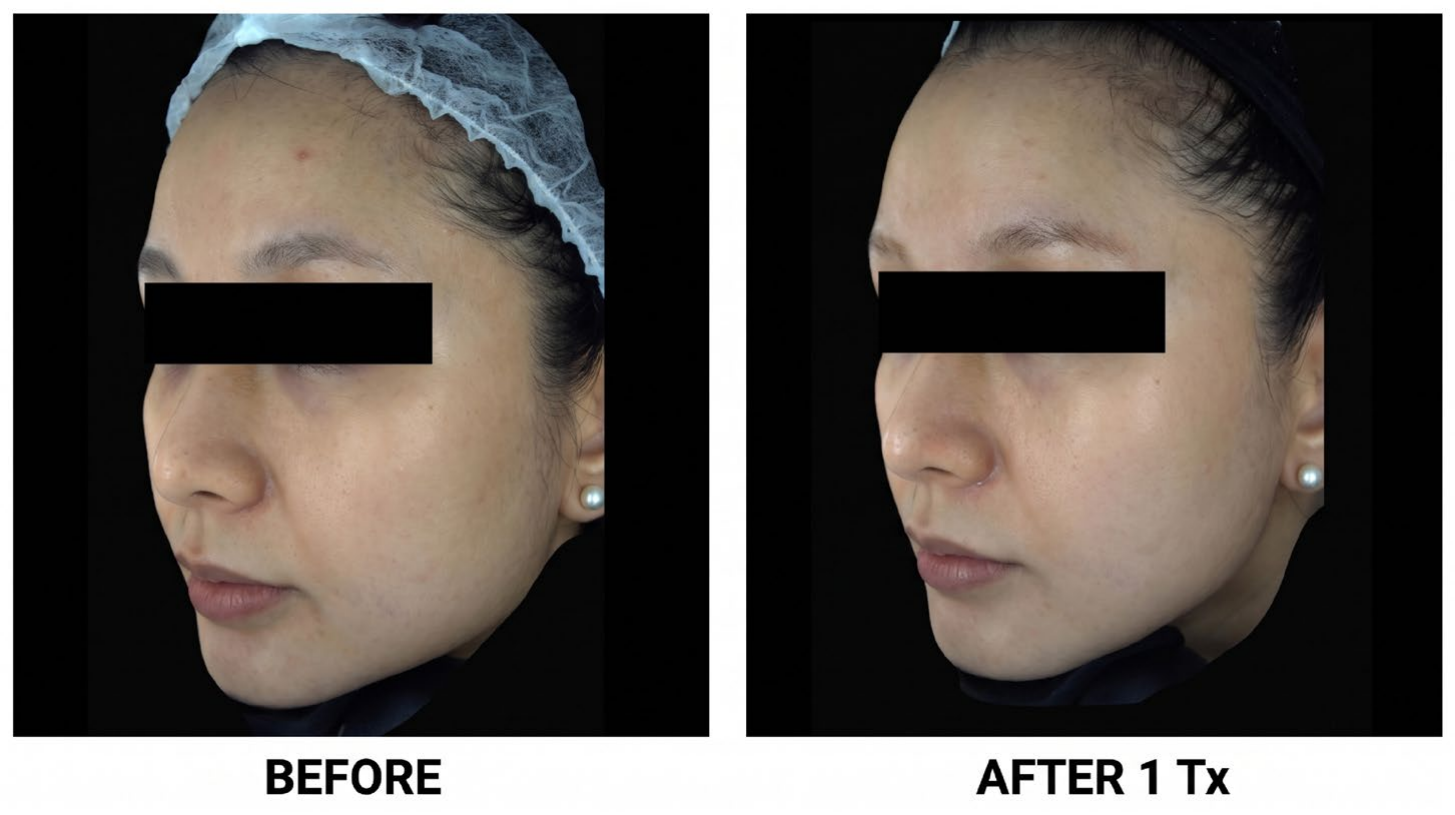
Case 2: Available clinical photographs before and after a single multiwavelength diode laser session, illustrating a qualitative comparison of jawline and nasolabial fold appearance. The eye region was masked for privacy.

The available photographs provide only a descriptive comparison following a single treatment session. Because the exact posttreatment assessment interval was not documented, the timing and durability of the observed changes cannot be established.

No direct measurements of tissue temperature, soft‐tissue contraction, or collagen remodeling were performed. Therefore, the observed appearance cannot be attributed to a specific thermal or structural mechanism.

### 3.3 Case 3: Male Facial Conditioning After Previous Biostimulatory Filler

A 38‐year‐old man with Fitzpatrick skin Type II underwent treatment for facial rejuvenation, with particular attention to the nasolabial folds and jawline.

The patient had received two syringes of calcium hydroxylapatite filler approximately 5 months before laser treatment. He subsequently underwent three multiwavelength diode laser sessions at 4‐week intervals.

The reported energy per visit was 60 kJ, comprising 10 kJ per facial side during stacked irradiation and 20 kJ per facial side during dynamic irradiation.

The available clinical photographs suggested improvements in facial tonal evenness, visible erythema, nasolabial fold appearance, and jawline definition following the treatment course (Figure 4).

**FIGURE 4:**
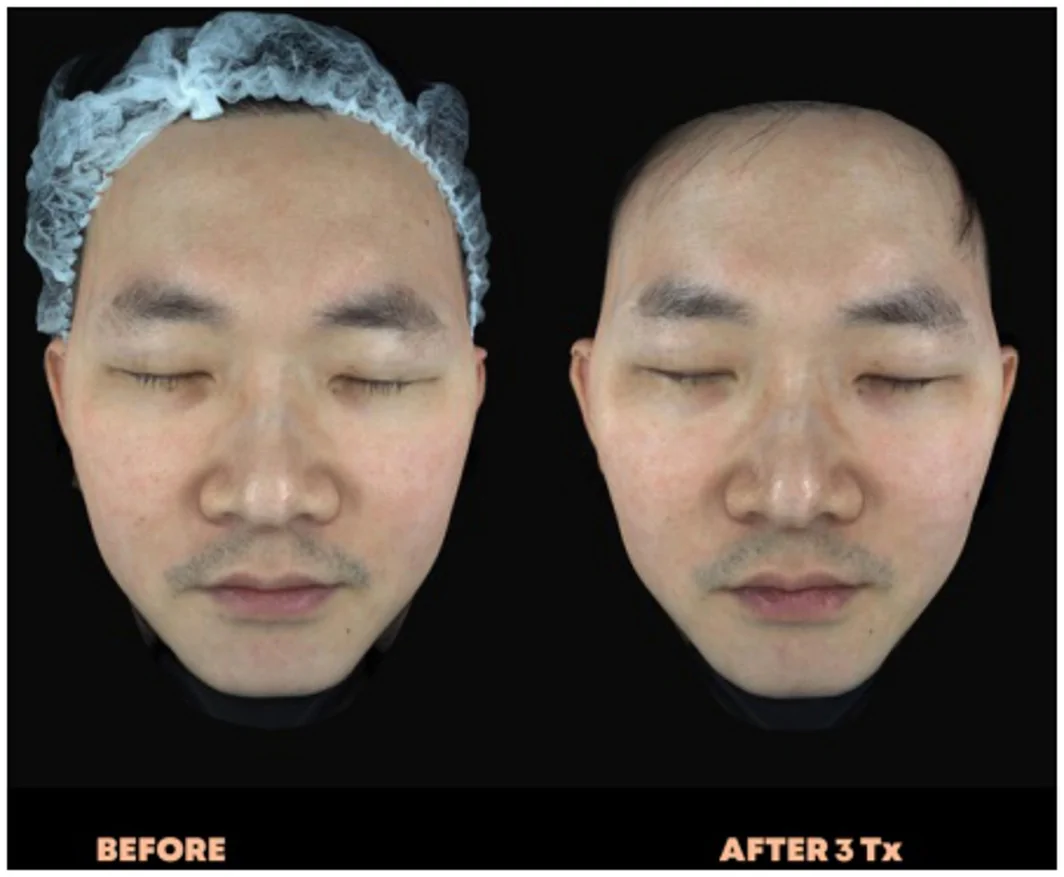
Case 3: Available clinical photographs before treatment and after three multiwavelength diode laser sessions, illustrating a qualitative comparison of facial appearance. The patient had received calcium hydroxylapatite filler approximately 5 months before laser treatment.

Previous calcium hydroxylapatite filler treatment represents a potential confounding factor. Because the patient received filler before the laser treatment course, the relative contributions of the previous intervention and subsequent laser sessions cannot be determined.

No measurements were performed to evaluate interactions between laser exposure and the previously injected material.

## 4 Discussion

This three‐patient case series describes preliminary clinical observations following multiwavelength diode laser treatment for different facial aesthetic concerns. The available photographs suggested changes in facial tonal evenness, erythema, pigmentation, nasolabial fold appearance, and jawline definition.

In Case 1, three‐dimensional skin‐analyzer measurements demonstrated changes in several redness and pigmentation parameters. However, the findings were derived from a small, uncontrolled, and heterogeneous series.

The observed outcomes do not establish treatment efficacy. Furthermore, the absence of direct thermal or histological measurements prevents conclusions regarding depth‐specific or multilayer tissue effects.

Previous investigations provide preliminary evidence for the clinical application of diode laser systems in facial rejuvenation.

Jo et al. reported clinical improvement and histological increases in collagen and elastic bundles following five treatment sessions with a combined 755‐, 810‐, and 1064‐nm laser [1].

Voravutinon et al. [2] investigated an 810‐ and 940‐nm diode laser for facial skin tightening.

These studies provide a rationale for further investigation but do not establish equivalent outcomes across different devices or treatment protocols. Differences in wavelength combinations, energy delivery, treatment parameters, and study design limit direct comparisons with the present case series.

Thermal stimulation and collagen remodeling represent possible biological mechanisms underlying nonablative laser rejuvenation.

Previous research using a 1064‐nm Nd:YAG laser demonstrated erythema reduction and histological evidence of new collagen formation [3]. Experimental research in a mouse model of rosacea‐like lesions also demonstrated changes in erythema and collagen‐related markers following 1064‐nm laser exposure [8]. These findings provide indirect mechanistic context rather than direct evidence for the present treatment protocol.

The combination of 755‐, 810‐, and 1064‐nm wavelengths may produce different patterns of optical absorption and energy deposition. However, tissue temperature, optical penetration, thermal distribution, and histological changes were not directly measured in this study.

Therefore, depth‐specific heating, cumulative bulk heating, and remodeling of individual tissue compartments remain proposed mechanisms rather than demonstrated treatment effects.

Changes in erythema and pigmentation were observed descriptively in Case 1. The skin‐analyzer outputs demonstrated decreases in redness quantities, areas, and percentages across the recorded severity categories. Pigmentation quantities and areas also decreased across the recorded categories, although the mild pigmentation percentage increased.

Previous investigations have reported erythema reduction following nonablative 1064‐nm Nd:YAG laser treatment [3, 8]. However, the present study did not directly assess vascular changes, melanin content, inflammatory markers, or tissue remodeling. The biological basis of the observed changes therefore remains uncertain.

The inclusion of patients with Fitzpatrick skin Type IV is clinically relevant when considering potential pigmentary complications. However, the available source material did not contain systematic adverse‐event assessments or sufficiently detailed posttreatment surveillance to establish the incidence of burns, pigmentary changes, or post‐inflammatory hyperpigmentation.

Safety in patients with darker skin types cannot be established from this limited series.

Apparent changes in jawline definition and nasolabial fold appearance were observed in Cases 2 and 3. However, these changes were evaluated using available photographs without objective measurements of facial volume, tissue thickness, or contour displacement.

Consequently, the findings cannot establish facial fat reduction, tissue contraction, or structural remodeling.

In Case 3, previous calcium hydroxylapatite filler treatment represents an additional confounding factor. The observed changes cannot be attributed exclusively to laser treatment, and no interaction between the laser and previously injected filler was directly investigated.

Adjunctive polynucleotide injections represent an important confounding factor in Case 1.

Previous reviews have discussed the use of polynucleotides in aesthetic medicine and their potential effects on skin quality [9, 10].

Because injections were administered after each laser session, the findings in this patient represent observations following a combined treatment regimen.

The independent contribution of the diode laser cannot be determined, and the results should not be interpreted as evidence of laser monotherapy efficacy.

This study has several important limitations.

First, the sample consisted of only three patients with heterogeneous aesthetic indications, and no control group was included. Adjunctive polynucleotide injections in Case 1 and previous calcium hydroxylapatite filler treatment in Case 3 prevent attribution of the observed outcomes exclusively to laser treatment.

Second, the photographic assessment intervals were not consistently documented, and objective skin‐analyzer measurements were available for only one patient. The clinical interpretation of the device‐specific scoring system was not established. No comparative statistical analysis, objective facial volume measurements, or long‐term durability assessments were performed.

Third, although the available records permitted reconstruction of the arithmetic distribution of the reported 40‐kJ and 60‐kJ treatment energies, their relationship to the actual delivered optical energy could not be independently verified. Important technical information, including the manufacturer and model, wavelength‐specific output power, emission mode, wavelength delivery sequence, spot size, handpiece configuration, treatment area, scanning speed, pass overlap, cooling method, and treatment duration was unavailable in the supplied material. These limitations restrict treatment reproducibility and prevent reliable characterization of the delivered thermal dose.

Fourth, the study did not include direct measurements of tissue temperature, optical penetration, thermal distribution, or histological changes. Therefore, the proposed depth‐specific heating and tissue‐remodeling mechanisms remain unconfirmed.

Finally, systematic adverse‐event data were not available, and the incidence of erythema, edema, burns, pigmentary changes, and post‐inflammatory hyperpigmentation could not be reliably determined.

Future prospective controlled studies should incorporate complete laser dosimetry, standardized treatment and imaging protocols, objective measurements of clinical outcomes, direct thermal assessments, structured adverse‐event monitoring, and longer‐term follow‐up.

## 5 Conclusion

This three‐patient case series describes preliminary photographic and skin‐analyzer observations following multiwavelength diode laser treatment for facial aesthetic concerns.

Although changes in facial appearance and selected device‐specific measurements were observed, the small sample size, absence of controls, concomitant or previous treatments, incomplete laser dosimetry, and limited follow‐up prevent conclusions regarding treatment efficacy or durability.

Depth‐specific heating and tissue remodeling were not directly demonstrated.

Further controlled investigations incorporating complete treatment parameters, objective outcome measurements, and systematic safety monitoring are required.

### 5.1 Safety Reporting

No serious adverse events were reported in the supplied clinical material. However, systematic adverse‐event data were not available, and the records did not provide detailed assessments of erythema, edema, burns, pigmentary changes, or post‐inflammatory hyperpigmentation.

Therefore, the absence of documented events should not be interpreted as evidence that no adverse events occurred.

The available data are insufficient to establish the safety profile of the treatment protocol.

## Author Contributions

All authors have reviewed and approved the article for submission. Conceptualization: Kyu‐Ho Yi. Methodology: Kyu‐Ho Yi, Jesse Chong Jia Xi. Data Curation: Jesse Chong Jia Xi. Formal Analysis: Jesse Chong Jia Xi. Writing – Original Draft Preparation: Jesse Chong Jia Xi. Writing – Review and Editing: Kyu‐Ho Yi, Jesse Chong Jia Xi. Visualization: Jesse Chong Jia Xi. Supervision: Kyu‐Ho Yi.

## Funding

The authors have nothing to report.

## Ethics Statement

The study received ethical approval from the Public Institutional Review Board (approval number: P01‐202607‐01‐121).

## Consent

Written informed consent for publication of the clinical photographs and relevant clinical information was obtained from all three patients.

## Conflicts of Interest

The authors declare no conflicts of interest.

## Data Availability

The data supporting the findings of this study are available from the corresponding author upon reasonable request, subject to applicable ethical and privacy restrictions.
